# Biomorphoelasticity alone: limitations in modeling post-burn contraction and hypertrophy without finite strains

**DOI:** 10.1007/s10237-025-01969-0

**Published:** 2025-06-05

**Authors:** Ginger Egberts, Fred Vermolen, Paul van Zuijlen

**Affiliations:** 1 Computational Mathematics Group, Department of Mathematics and Statistics, University of Hasselt, Diepenbeek, Belgium; 2 Dutch Burns Foundation, Beverwijk, The Netherlands; 3 Data Science Institute (DSI), University of Hasselt, Hasselt, Belgium; 4 Burn Center, Department of Plastic, Reconstructive and Hand Surgery, Red Cross Hospital, Beverwijk, The Netherlands; 5 Department of Plastic, Reconstructive and Hand Surgery, Amsterdam Movement Sciences, Amsterdam UMC, Location VUmc, Amsterdam, The Netherlands; 6 Pediatric Surgical Centre, Emma Children’s Hospital, Amsterdam UMC, Location AMC and VUmc, Amsterdam, The Netherlands

## Abstract

We present a continuum hypothesis-based two-dimensional biomorphoelastic model describing post-burn scar hypertrophy and contraction. The model is based on morphoelasticity for permanent deformations and combined with a chemical-biological model that incorporates cellular densities, collagen density, and the concentration of chemoattractants. We perform a sensitivity analysis for the independent parameters of the model and focus on the effects on the features of the post-burn dermal thickness given a low myofibroblast apoptosis rate. We conclude that the most sensitive parameters are the equilibrium collagen concentration, the signaling molecule secretion rate and the cell force constant, and link these results to stability constraints. Next, we observe a relationship between the simulated contraction and hypertrophy and show the effects for significant variations in the myofibroblast apoptosis rate (high/low). Our ultimate goal is to optimize post-burn treatments, by developing models that predict with a high degree of certainty. We consider the presented model and sensitivity analysis to be a step toward their construction.

## Introduction

Burn wounds are painful, and the healing process can take a long time. Wound healing is complicated, and deep burns often result in a scar. These scars can become hypertrophic, often a consequence of abnormalities in ‘normal’ wound healing. Hypertrophic scars (HSs) are generally thick, firm, dark red, and irregular. Patients with HSs can suffer enormously from itching and pain. As a result, patients with HSs not only experience a physical burden but also a psychological one.

Burn wound healing occurs in three overlapping phases, starting with the inflammatory response. During this response, signaling molecules are released that stimulate surrounding cells to migrate to the wound area. The migration of these cells is the beginning of the second phase, proliferation. Fibroblasts, which produce collagen, may differentiate into myofibroblasts under the influence of signaling molecules. Myofibroblasts further facilitate the contraction of the surrounding tissue. The spongy granulation tissue is eventually converted into scar tissue (remodeling), a process involving the reorganization of collagen fibers, by which the scar matures and gains strength. Myofibroblasts undergo apoptosis during a normal healing process (programmed cell death), however in HSs, myofibroblasts persist. The result is excessive extracellular matrix component deposition and tissue contraction. We note that the first phase of normal wound healing, hemostasis, is less significant in burn wound healing because small blood vessels usually close when burned.

Although HSs have been studied extensively, these scars still occur. Of course, HSs and their treatment are studied from a medical and biological perspective, which has led to classical therapies. However, it is only sometimes sure that recurrent hypertrophy is excluded. Next to studying HSs *in vitro*, *in vivo* and *ex vivo*, *in silico* provides an alternative strategy, using mathematical modeling. Previously, Koppenol developed two models: a model for the formation of hypertrophy (H model, Koppenol et al. 2017) and a model for the formation of contraction (C model, Koppenol and Vermolen 2017). The C model incorporates morphoelasticity, while the H model does not. The C model’s computational domain is parallel to the skin, while the H model’s is perpendicular. Both models showed a good match with clinical measurements. Specifically, the H model’s predictions of dermal thickness over time closely aligned with clinical measurements of hypertrophic and normal scars, as shown in Fig. 6 from Koppenol et al. (2017). Koppenol’s C model demonstrated good agreement with clinical data, as evidenced by the comparison of simulated wound surface area reduction to measurements from El Hadidy et al. (June 1994), shown in Fig. 5 in Koppenol and Vermolen (2017).

Post-burn contractures and hypertrophic scars frequently coexist and significantly impact patient quality of life; however, they can also occur independently. Therefore, developing models that can simulate both features and capture their complex interplay and individual manifestations is essential. While morphoelastic models have been used to simulate permanent deformations in post-burn tissue, their applicability to hypertrophic scar formation has not been fully explored. In this study, we investigate whether a morphoelastic framework can represent key aspects of hypertrophy, and we perform a sensitivity analysis to understand the model’s behavior and parameter responses.

We integrated elements of both the H and C models. Specifically, we utilized the morphoelasticity framework of the C model in the domain of the H model. We adopted the parameter values and perpendicular computational domain of the H model. To complete the model, we incorporated the additional parameters from the C model ($\rho _t$, $\mu _1$, $\mu _2$, and $\zeta$), which were necessary due to the inclusion of morphoelasticity. The parameters $\rho _t$, $\mu _1$, $\mu _2$, and $\zeta$ retained the values used in Koppenol’s original C model.

## The mathematical model

The biomorphoelastic model can simulate a permanent deformation resulting from post-burn contraction and hypertrophy. In short, the model comprises a system of coupled, nonlinear partial differential equations representing biochemical quantities, such as cell densities and concentrations of signaling molecules and collagen, as well as the displacement velocity and the effective strain (mechanics). The interaction between the model variables leads to contraction and hypertrophy of the damaged skin that we measure with the displacement ($\varvec{u}$).

The biochemical variables are the fibroblast density (*N*), the myofibroblast density (*M*), the signaling molecule density (*c*), and the collagen density ($\rho$). The equations are based on conservation laws and have the following form

1 $$\begin{aligned} \frac{\text {D}z}{\text {D}t} + z[\nabla \cdot \varvec{v}] = -\nabla \cdot \textbf{J}_z + R_z, \end{aligned}$$

with $z\in \{N,M,c,\rho \}$. Here, $\varvec{v}$ is the displacement velocity, i.e., the velocity with which each point in the tissue displaces because of contraction and hypertrophy. The model involves the material time derivative $\frac{\textrm{D}z}{\textrm{D}t} = \frac{\partial z}{\partial t} + \varvec{v}\cdot \nabla z$ and passive convection $z(\nabla \cdot \varvec{v})$, $z\in \{N,M,c,\rho ,\varvec{v}\}$. These concepts are introduced because the computational domain is subject to displacement because of the forces exerted by the cells.

In equation (1), $\textbf{J}_z$ and $R_z$ denote the flux and the biochemical kinetics of *z*, respectively, and have the following functional forms:

2 $$\begin{aligned} \textbf{J}_N&= -D_n(N+M)\nabla N + \chi N \nabla c, \end{aligned}$$

3 $$\begin{aligned} \textbf{J}_M&= -D_n(N+M)\nabla M + \chi M \nabla c, \end{aligned}$$

4 $$\begin{aligned} \textbf{J}_c&= -D_c\nabla c. \end{aligned}$$

Here, $D_{n}$ is the diffusion constant for the fibroblasts and the myofibroblasts, $D_c$ the signaling molecule diffusion constant, and $\chi$ is the chemotactic parameter. Equations (2) and (3) represent migration toward the gradient of the signaling molecules (Postlethwaite et al. 1987; Boon et al. 2016; Dallon et al. 2001) and cell density-dependent Fickian diffusion (random walk). Collagen molecules are assumed to have no active transport because they are large, reducing their diffusivity. Since collagen is extracellular, it is, next to diffusion, not subject to other active migration mechanisms. Hence, $\textbf{J}_{\rho } = \textbf{0}$.

The fibroblast proliferation depends on a generic chemokine via an activator/inhibitor mechanism (Murray 2011). Furthermore, myofibroblast differentiation only proceeds in the presence of the chemokine (Tomasek et al. 2002). Apoptosis is taken into account via a linear relation. The myofibroblast dynamics are similar, except that it is assumed that myofibroblasts proliferate only in the presence of the signaling molecules. It is well-known that myofibroblasts proliferate much less than fibroblasts (Vaughan et al. 2014), so we use a distinct cell proliferation rate for the cells:

5 $$\begin{aligned} R_N&= r_n \left[ 1+\frac{r^{\text {max}}c}{a_c^{I}+c} \right] [1-\kappa (N+M)] N^{1+q} - k_1 cN - \delta _n N, \end{aligned}$$

6 $$\begin{aligned} R_M&= r_m \left[ \frac{[1 + r^{\text {max}}]c}{a_c^{I}+c} \right] [1-\kappa (N+M)] M^{1+q} + k_1 cN - \delta _m M. \end{aligned}$$

Here, $r_{n/m}$ are the proliferation rates of the fibroblasts and the myofibroblasts, respectively, $r^{\max }$ and $a_c^{I}$ are the proliferation enhancement factor, and half-maximal enhancement factor, respectively. Further, $\kappa$ is the crowding factor (Vande Berg et al. 1989), *q* is a constant used to model equilibrium, $k_1$ is the differentiation factor (Tomasek et al. 2002), and $\delta _{n/m}$ are the fibroblast and myofibroblast apoptosis rates, respectively.

The signaling molecule and collagen kinetics describe secretion by the fibroblasts and myofibroblasts (Barrientos et al. 2008; Baum and Arpey 2006), where signaling molecules enhance the collagen secretion (Ivanoff et al. 2005). Decay of signaling molecules and collagen is because of cleavage by matrix metalloproteinases (MMPs) (Mast and Schultz 1996; Sternlicht and Werb 2001). The MMP concentration is assumed to be proportional to the cell density of the fibroblasts and myofibroblasts, and the concentration of both the collagen molecules and the signaling molecules (Lindner et al. 2012):

7 $$\begin{aligned} R_c&= k_c \left[ \frac{c}{a_c^{II} + c} \right] [N + \eta ^I M] - \delta _c \frac{[N + \eta ^{II}M]\rho }{1 + a_c^{III}c} c, \end{aligned}$$

8 $$\begin{aligned} R_\rho&= k_\rho \left[ 1 + \left[ \frac{k_\rho ^{\text {max}}c}{a_c^{IV} + c} \right] \right] [N + \eta ^I M] - \delta _\rho \frac{[N + \eta ^{II}M]\rho }{1 + a_c^{III}c} \rho . \end{aligned}$$

Here, $k_{c/\rho }$ are the signaling molecule and collagen secretion rates, and $a_c^{II/IV}$ their inhibition signaling molecule concentrations. Further, $k_\rho ^{\max }$ is the collagen secretion enhancement factor (Ivanoff et al. 2005), and $a_c^{III}$ is the signaling molecule concentration inhibiting MMP release (Overall et al. 1991). The parameters $\eta ^I$ and $\eta ^{II}$ represent the proportions of myofibroblasts in the maximum net secretion rates of the signaling molecules/collagen and MMPs, respectively. Further, $\delta _{c/\rho }$ are the coefficients describing decay because of MMP cleavage. The generic MMP affecting the above reaction kinetics is always assumed to be at a local equilibrium concentration. This modeling choice has avoided even more complexity and additional unknown parameter values.

For the mechanics, we have the displacement velocity ($\varvec{v}$) and effective (remaining) Eulerian strain ($\varvec{\varepsilon }$). The Cauchy stress tensor $\sigma$ in the displacement velocity equation is related to the effective Eulerian strain and displacement velocity gradients by a visco-elastic constitutive relation. The body force $\textbf{f}$ is generated by a pulling force on the extracellular matrix by myofibroblasts, which is proportional to the product of the myofibroblast cell density and a function of the collagen concentration. The balance of momentum leads to

9 $$\begin{aligned} \rho _t \left( \frac{\text {D} \varvec{v}}{\text {D} t} + \varvec{v}[\nabla \cdot \varvec{v}] \right) = \nabla \cdot \sigma + \textbf{f} = \nabla \cdot \sigma + \nabla \cdot \left( \frac{\xi M\rho }{R^2+\rho ^2} \right) \textbf{I}. \end{aligned}$$

Here, $\rho _t$ represents the total mass density of the dermal tissues, $\xi$ is the generated stress per unit cell density, and *R* is the body force-inhibiting constant.

The visco-elastic constitutive relation follows the assumption from Ramtani (Ramtani 2004; Ramtani et al. 2002), which incorporates the dependence of the Young’s modulus of skin on the collagen density. From a mechanical point of view, the tissue is assumed to be isotropic and homogeneous, except for a dependency of the stiffness on the local collagen density:

10 $$\begin{aligned} \sigma = \mu _1\text {sym}(\nabla \varvec{v}) + \mu _2(\text {tr}(\text {sym}(\nabla \varvec{v}))\textbf{I}) + \frac{E\sqrt{\rho }}{1+\nu }\left[ \varvec{\varepsilon }+\text {tr}(\varvec{\varepsilon })\frac{\nu }{1-2\nu }\textbf{I}\right] , \end{aligned}$$

where $\mu _1$ and $\mu _2$ are the shear and bulk viscosities, $E\sqrt{\rho }$ represents Young’s modulus (stiffness), and $\nu$ is the Poisson’s ratio. Further, $\text {sym}(\nabla \varvec{v})=\frac{1}{2}(\nabla \varvec{v}+\nabla \varvec{v}^\text {T})$. Despite possibly large deformations in the tissue, linear elasticity is used to avoid the requirement of additional input parameters, of which the value is unknown or, at least, uncertain. We note that in Koppenol et al. (2017), Koppenol modeled the dermal layer as an isotropic compressible neo-Hookean solid. The isotropic assumption is a simplification, as skin is known to exhibit anisotropic behavior. However, this assumption was made to reduce model complexity in this initial study.

Permanent deformation is incorporated via morphoelasticity, of which the (multidimensional) derivation can be found in Hall (2008). The contraction of the tissue is modeled with a negative sign. It is assumed to be proportional to the product of the effective Eulerian strain, the cell densities, and a function of the collagen density. In particular, the contraction tensor depends on the product of the MMP and the signaling molecule concentrations. It is inversely proportional to the collagen density (note that the collagen density drops out because of the linear dependence of the equilibrium MMP concentration on the collagen density):

11 $$\begin{aligned}&\frac{\text {D}\varvec{\varepsilon }}{\text {D}t} + \varvec{\varepsilon }\,\text {skw}(\nabla \varvec{v}) - \text {skw}(\nabla \varvec{v})\varvec{\varepsilon } + (\text {tr}(\varvec{\varepsilon })-1)\text {sym}(\nabla \varvec{v}) \nonumber \\&= -\zeta \frac{[N+\eta ^{II}M]c}{1+a_c^{III}c}\varvec{\varepsilon }. \end{aligned}$$

Here, $\zeta$ is the rate of morphoelastic change, i.e., the rate at which the effective strain changes actively over time. Further, $\text {skw}(\nabla \varvec{v})=\frac{1}{2}(\nabla \varvec{v}-\nabla \varvec{v}^\text {T})$.

It is important to recognize that the model is a simplification of the complex biological processes involved in burn wound healing. As such, it may not fully capture all the factors that influence tissue thickness and wound contraction.

Parameter values

The model incorporates a range of parameters that influence the biochemical and mechanical processes of wound healing. The mean values of these parameters are summarized in Table 1 in the Appendix. These values were primarily derived from the previously validated H and C models developed by Koppenol and Vermolen (2017), Koppenol et al. (2017). However, it is important to note that a sensitivity analysis was conducted, during which 30 of the 35 model parameters were systematically varied by $\pm \{5, 10, 15\}\%$. These variation ranges were chosen to explore a moderate range of parameter perturbations while maintaining mathematical stability of the model. If the system is highly sensitive to a parameter and smaller variations are needed, this would be seen in the results of the sensitivity analysis.

The parameters $\rho _t, \mu _1,\mu _2$, and $\zeta$ were adopted directly from Koppenol and Vermolen (2017). These values were chosen based on their demonstrated ability to accurately represent the mechanical properties of skin tissue.

We left the values for five parameters content because of several reasons. Like before, we excluded variations in the Poisson’s ratio ($\nu$) (Egberts et al. 2022). The initial conditions for fibroblast cell and collagen densities are set to 20% of the equilibria densities. Therefore, these values vary together with the equilibria values. The constant *q* and the collagen secretion rate $k_\rho$ were treated as dependent parameters. These latter two values are computed by solving the equations for equilibria.

For a complete list of parameter values, including their units and descriptions, we refer to Table 1 in the Appendix.

The computational domain and the boundary conditions

**Fig. 1:**
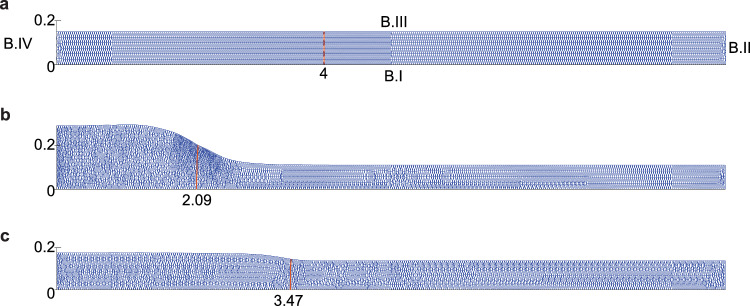
Example of the domain of computation at different times. The boundaries BI, B.II, B.III, and B.IV are shown in Fig.**a**. Further, each figure shows two ticks on boundary B.IV corresponding to $y=0$ and $y=0.2$ cm, and on boundary BI, the figures show the tick for the wound boundary in cm. Fig.**a** shows the mesh on day 0, Fig.**b** on the day when the hypertrophy is maximal, and Fig.**c** on day 365. The figures correspond to a simulation where the myofibroblast apoptosis rate is $\delta _m=0.06$/day (a high rate)

We define the two-dimensional computational domain by $\Omega _{\textbf{x}}$ and its boundary by $\partial \Omega _i$ with $i \in \{ I, II, III, IV \}$. Here $\partial \Omega _I$ represents the lower boundary where $y=0$, $\partial \Omega _{II}$ represents the right essential boundary at $x=10$, $\partial \Omega _{III}$ represents the upper boundary (that moves because of hypertrophy), and $\partial \Omega _{IV}$ represents the symmetrical boundary at $x=0$, see Fig. 1a. For the chemicals, we use the following boundary conditions for all time *t* and all

12 $$\begin{aligned} \textbf{x}\in \partial \Omega _{II}&: \quad N(\textbf{x};t)=\overline{N},\quad M(\textbf{x};t)=\overline{M},\quad \text {and}\quad c(\textbf{x};t)=\overline{c}, \end{aligned}$$

13 $$\begin{aligned} \textbf{x}\in \partial \Omega _{I,III,IV}&: \quad \textbf{J}_{N/M/c} \cdot \textbf{n} = 0, \end{aligned}$$

where $\textbf{n}$ is the outward pointing normal vector.

Let $\varvec{v}=[v_1,v_2]^T$ and $$\sigma =\begin{bmatrix}\\ \sigma _{.1}& \sigma _{.2}\\ \\ \end{bmatrix}$$, for the mechanics; we use for all time *t*

14 $$\begin{aligned} \textbf{x}\in \partial \Omega _{I}&: \quad v_2(\textbf{x};\,t)=0 \quad \text {and}\quad (\sigma _{.1} \cdot \textbf{n})\cdot \tau =0, \end{aligned}$$

15 $$\begin{aligned} \textbf{x}\in \partial \Omega _{II,IV}&: \quad v_1(\textbf{x};\,t)=0\quad \text {and}\quad (\sigma _{.2} \cdot \textbf{n})\cdot \tau =0, \end{aligned}$$

16 $$\begin{aligned} \textbf{x}\in \partial \Omega _{III}&: \quad (\sigma \cdot \textbf{n})\cdot \tau = 0, \end{aligned}$$

where $\tau$ is the tangential vector. These boundary conditions imply that boundary B.I is free to move in the direction of the *x*-axis, while it is fixed in the direction of the *y*-axis. For boundaries B.II and B.IV, the boundary conditions imply that these boundaries are free to move in the direction of the *y*-axis while they are fixed in the direction of the *x*-axis. The boundary condition for boundary B.III implies that this boundary is free to move in any direction. For clarification, we show in Figure 1 the domain of computation at different times, in which the wound edge is also visible.

The initial conditions

The initial conditions represent the start of proliferation. During inflammation, signaling molecules stimulate fibroblast migration; however, myofibroblast differentiation does not happen yet; and hence, the computational domain is not yet subject to displacement. Hence, the initial conditions for the myofibroblast cell density, the displacement velocity density, and the effective Eulerian strain density are zero.

The initial fibroblast and collagen densities were adopted from Koppenol and Vermolen (2017); Koppenol et al. (2017). These values represent the cell and matrix composition of the tissue immediately following inflammation. Specifically, the initial fibroblast and collagen densities were set to 20% of their respective equilibrium values ($0.2\cdot \overline{N}$ and $0.2\cdot \overline{\rho }$). This choice reflects the assumption that the wound starts with a reduced cellular and matrix density relative to healthy tissue. The initial concentration of signaling molecules was adopted from Olsen et al. (1995), who provided a rationale for this value based on the concentration of platelet-derived growth factor (PDGF) in blood. Olsen et al. noted that PDGF, a key signaling molecule involved in wound healing, is stored within platelets at concentrations of 15-50 ng/ml. They reasoned that the concentration of PDGF released at the wound site by platelets and activated inflammatory cells would not exceed these levels. Based on this, Olsen et al. (1995) proposed an initial concentration of 10 ng/ml for PDGF, which was used as the basis for the initial signaling molecule concentration in our model. To align with the units used in our model, this value was converted to $10^{-8}$ g/cm$^3$. This initial concentration of signaling molecules represents the presence of these factors in the early inflammatory phase, where they play a crucial role in initiating fibroblast migration and other wound healing processes.

In addition to the initial values, the spatial distribution of these species is also important. The initial conditions lead to a steep drop in values at the wound boundary. To account for this, we define the wound boundary steepness size by s = 0.5 cm, which accounts for the species’ slope on the wound boundary. We model the slopes of the species with sine functions, similar to what Koppenol and Vermolen (2017); Koppenol et al. (2017) did.

## Implementation

We solve the equations using the finite element method and implement the solution method in MATLAB. For a detailed description of the solution to the equations, we refer to the appendix in Koppenol (2017). We subdivide the computational domain into a finite number of nonoverlapping triangles and choose the linear Lagrangian basis functions for the finite-dimensional subspace. We approximate the integrals over the interior of the elements by a Newton-Cotes rule based on linear basis functions. We solve the Galerkin equations using backward Euler time integration, and we use a monolithic approach with inner Picard fixed point iterations to account for the nonlinearity of the equations. In all simulations, the dimension $\textbf{x}$ is in centimeters and *t* in days.

*Remeshing* In the simulations, the computational domain becomes ‘hypertrophic’. We approximate the local displacements of the dermal layer $(\varvec{u})$ with

17 $$\begin{aligned} {\varvec{u}}(\textbf{x};\,t+\Delta t) \approx {\varvec{u}}(\textbf{x};\,t) + {\varvec{v}}(\textbf{x};\,t)\Delta t. \end{aligned}$$

For the displacement we use the initial condition $\varvec{u}(\textbf{x};\,0) = 0,\,\forall \textbf{x}\in \Omega _{\textbf{x},0}$. Further, we update the mesh (triangulation) in every time integration step, and we determine the quality of this updated mesh by computing

$$\min _{e_k}\left| \textbf{J}_{e_k}\right| /\max _{e_k}\left| \textbf{J}_{e_k}\right| ,\quad e_k\in \Omega ,$$

with $\textbf{J}$ the Jacobian of the transformation of element $e_k$ to a reference element which is a unit right triangle. The above expression reflects the ratio between the areas of the smallest and largest triangular element. In case $\min _{e_k}\left| \textbf{J}_{e_k}\right| /\max _{e_k}\left| \textbf{J}_{e_k}\right| <0.5$ then the mesh quality is deemed to be too low, and hence, we perform remeshing. We create a new grid and triangulation.

During remeshing, we remesh the previous mesh such that the current time iteration converges with an acceptable mesh quality. The Picard iterations might not converge after remeshing, in which case we decrease the timestep to 50% of its current value and move back to the previous time iteration.

*Control of the timestep* Regardless of remeshing, if the Picard iterations do not meet the convergence criterion within six iterations, we decrease the timestep to 80% of its current value and restart the Picard iterations. Otherwise, we increase the timestep by a factor of 1.1, with a maximum $\Delta t_{\max }$ depending on the change in the domain’s height in the center at the symmetrical axis. Initially, the maximal timestep is $\Delta t_{\max ,1}=0.05$ day for half a day, after which $\Delta t_{\max ,2}=0.5$ day. If the domain’s height decreases (assuming the maximum is reached), the maximal timestep increases by 10% if the Picard iterations converge in two iterations. We start with an initial timestep of $\Delta t=0.05$ day.

In some cases, we need more control. These are the cases when the Picard iterations do not converge after remeshing. In such case, the code goes back in time five time iterations and halves the timestep.

## Sensitivity analysis

We perform a sensitivity analysis to determine the extent to which hypertrophy is effected by variations in parameter values. This way, we know which parameters are so-called ‘sensitive’.

We vary 30 independent parameter values out of the 35 model parameters to study their sensitivity. The dependent parameters are the constant *q*, the collagen secretion rate $k_\rho$, and other parameters that we keep constant or let depend on other parameter values are the initial fibroblast cell and collagen densities and the Poisson’s value $\nu$.

We vary the parameter values by increasing or decreasing the average values by $\pm \{5,10,15\}\%$ so that we run a total of 362 simulations: 6 variations $\times$ 30 parameters + a single control run for both $\delta _m=0.02$/day and $\delta _m=0.06$/day, and we respect the model’s stability constraint $k_c\le \delta _c a_c^{II}\overline{\rho }$ (Egberts et al. 2023). Table 1 in the Appendix 7 shows the average parameter values. The results show the *maximum of the tissue thickness in the center of the wound* (TH$_{\max }$) in a time of one year, the *day on which the thickness reaches the maximum* (TH$_{\text {day}}$, i.e., the day after which the thickness reduces), and the *tissue thickness in the center of the wound on day 365* (TH$_{end}$).

Each parameter $i\in \{D_n,\dots ,\tilde{c}\}$ has a *z*-score for values in $r\in \{\text {TH}_{\max },\text {TH}_{\text {day}},\text {TH}_{\text {end}}\}$ and variation $j\in \{\pm$ 5, 15, 25%} defined by $z_{ij}^r = (x_{ij}^r -\overline{x}_j^r)/s_{x_j^r}$. Here $\overline{x}_j^r$ is the sample mean, and $s_{x_j^r}$ is the sample standard deviation. We measure the sensitivity by the sum of the absolute values of the *z*-scores:

18 $$\begin{aligned} \mathcal {S}_i^r = \sum _{j} \left| z_{ij}^r \right| , \end{aligned}$$

where $z_{ij}^r$ is the *z*-score of the data in *r* for parameter *i*.

**Fig. 2:**
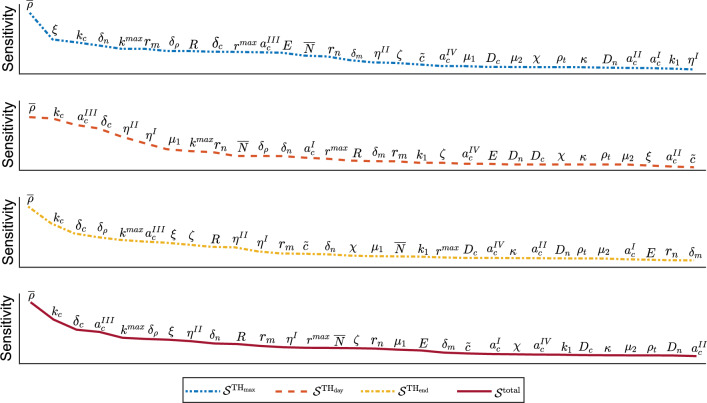
Parameter sensitivity in terms of *z*-scores for $\delta _m=0.02$/day. From top to bottom, the blue two-point-dashed curve shows the parameter sensitivity for the maximum tissue thickness ($\mathcal {S}^{\text {TH}_\text {max}}$), the orange dashed curve for the day of maximum tissue thickness ($\mathcal {S}^{\text {TH}_\text {day}}$), the yellow one-point-dashed curve for the tissue thickness on day 365 ($\mathcal {S}^{\text {TH}_\text {end}}$), and the dark red solid curve shows the total sensitivity ($\mathcal {S}^\text {total}$)

Figure 2 shows a visualization of the sensitivity for all varied parameter values given $\delta _m=0.02$/day (a low myofibroblast apoptosis value). The corresponding *z*-scores are shown in Table 2 in the Appendix 7.

In this overview, we see that the equilibrium collagen concentration ($\overline{\rho }$) scores highest in sensitivity for all outcomes (the maximum tissue thickness, the day of maximum tissue thickness, the tissue thickness after one year, and the total score). The relatively large jump in $\mathcal {S}^{\text {TH}_\text {max}}$ scores to the cell force constant ($\xi$) and the signaling molecule secretion rate ($k_c$) shows that the equilibrium collagen concentration is an outlier in sensitivity. The cell force constant has a considerable influence on hypertrophy during healing, while it hardly influences the timing of hypertrophy (visible in the orange dashed curve) and, therefore, scores much lower in the total score. We further see that signaling molecule secretion scores high in sensitivity on all outcomes. The course of the curve representing the day of maximum tissue thickness is interesting. Here, we see a group of parameters (collagen and signaling, and factors $\eta ^{I/II}$) that has a more extensive spread in all other outcomes. The relatively large sensitivity indicates, according to the model, that signaling has an important influence on the timing of hypertrophy, together with the portions of myofibroblasts that secrete signaling and collagen molecules and MMPs. These variables are related to the stability constraint $k_c\le \delta _c a_c^{II}\overline{\rho }$ (Egberts et al. 2023). In the control run, where we do not vary parameter values, the constraint is of order $k_c=\mathcal {O}(10^{-13})\le \delta _c a_c^{II} \overline{\rho }=\mathcal {O}(10^{-4})\mathcal {O}(10^{-8})\mathcal {O}(10^{-1})=\mathcal {O}(10^{-13})$. Hence, if we increase the signaling molecule secretion rate and decrease the signaling molecule decay rate and the equilibrium collagen concentration, then the undamaged equilibrium state might become unstable (Egberts et al. 2023). Given that $\overline{\rho }$ is of order $\mathcal {O}(10^{-1})$, it is not surprising that these variations approach the stability limit more quickly than variations in the signaling molecule secretion and decay rate values. Where the simulations with low equilibrium collagen showed many time steps where remeshing was needed, *after* the maximum of the tissue thickness was reached, the simulations with high ‘signaling secretion and decay’ showed many time steps with remeshing, *before* maximum tissue thickness was reached. The biology of the model can explain the latter. The signaling molecules stimulate fibroblasts to differentiate (Eq. (5)) and are primarily present before the maximum tissue thickness is reached. Therefore, increased signaling molecule secretion and decreased decay result in an abundance of myofibroblasts that produce collagen and contract the tissue, resulting in more intense hypertrophy during signaling.

Summarized, we see that collagen significantly influences all outcome measures. Further, we see the distinction between the cells and signaling molecules: the cells significantly influence maximum hypertrophy, while signaling molecules significantly influence the timing of hypertrophy and the remaining hypertrophy after one year. From a mechanical point of view, sensitivity is caused by the pulling forces of the cells rather than by tissue properties (Young’s Modulus, (shear/bulk), viscosities, and morphoelastic change). Regarding the mechanical parameters, we see that the Youngs Modulus (*E*) influences the maximum hypertrophy the most, while the shear viscosity ($\mu _1$), the timing, and the morphoelastic change ($\zeta$) the remaining hypertrophy.

We note that the myofibroblast apoptosis rate scores low when $\delta _m=0.02$/day. If the myofibroblast apoptosis rate is three times as fast ($\delta _m=0.06$/day), then fluctuations in this order will score higher in sensitivity.

Now that we know the sensitivity of variations in parameter values to a low myofibroblast apoptosis rate, we do not yet know the effect of these variations. To reduce hypertrophy and its timing, one needs to increase the collagen concentration (figures not shown). Significant deviations imply that low collagen concentrations ensure a more protracted hypertrophy process. This conclusion is in line with what parameter *E* shows: a lower Young’s modulus increases hypertrophy. This effect makes sense, as tissues with low stiffness offer less resistance to deformation. Interestingly, hypertrophy increases for lower collagen decay rates. When collagen decay is low, more collagen remains present. However, the order of the decay value is $\mathcal {O}(10^{-6})$ cm$^6$/(cells g day). Therefore, the variations in the equilibrium collagen concentration significantly influence tissue thickness. Hypertrophy is further effectively reduced by decreasing fibroblast apoptosis ($\delta _n$), increasing collagen secretion by signaling molecules, and lowering stress. Furthermore, elevated signaling protracts the hypertrophy process, which aligns with the fact that excessive inflammation can lead to delayed healing (Pierce August 2001).

When targeting hypertrophy (when myofibroblasts persist), the question arises whether we can influence the parameter values. Given the equilibrium collagen concentration sensitivity, our results show that increasing the collagen concentration is most effective, for example, using a full-thickness skin graft. Our simulation, where we increase the initial collagen concentration in the wound with 50% of the equilibrium collagen concentration instead of 20%, shows that the maximum tissue thickness reduces with 0.04 cm and the tissue thickness on day 365 with 0.009 cm. If we set the initial collagen concentration to 100%, then the maximum tissue thickness reduces by 0.14 cm and the tissue thickness on day 365 to 0.03 cm.

**Fig. 3:**
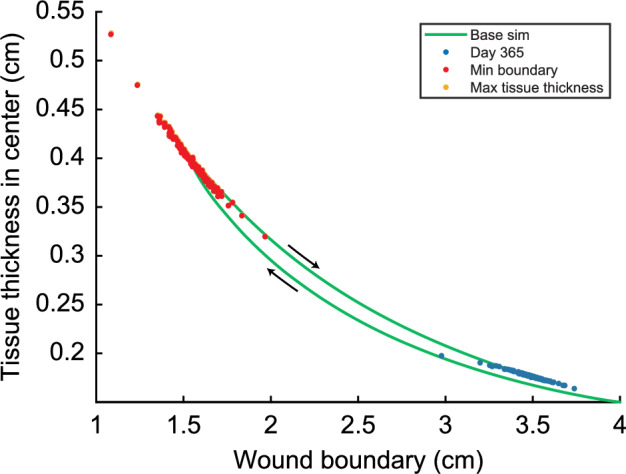
Relationship between the wound boundary and the maximum of the tissue thickness in the center (TH$_{\max }$) in a time of one year for $\delta _m=0.02$/day. Here, the yellow dots show the relationship between TH$_{\max }$ and the wound boundary when the TH$_{\max }$ is reached, the red dots show the relationship between the minimum of the wound boundary (maximum contraction) and the TH$_{\max }$ when the minimum of the wound boundary is reached, and the blue dots show the relationship between the tissue thickness on day 365 and the boundary of the wound on day 365. The yellow dots are hardly visible because these almost collapse with the red dots

Finally, we observe a relationship between the model’s predicted contraction and hypertrophy, shown in Fig. 3. In Fig. 3, a low value for the wound boundary corresponds to a high degree of contraction.

The green solid curve shows the relationship between the wound boundary and the tissue thickness in the center over time for the control run. The maximal contraction is 61.3% when the wound boundary is 1.5474 cm on day 63. By day 63, the tissue thickness has increased to 0.3958 cm. After maximal contraction, the tissue thickness increases slightly to 0.3966 cm while the wound boundary moves to 1.5504 cm (retraction) on day 65. From day 65, the tissue thickness decreases to 0.176 cm, and the wound retracts to 3.48 cm. Note that there is a slight difference between the day of maximal contraction and the day of maximal tissue thickness.

Instead of showing the curves for all simulations, we show three data points from each curve of the other 180 simulations in Figure 3. The yellow dots show the datapoint where the tissue thickness is maximal, and the red dots show where the contraction is maximal. Given that the yellow and red dots are very close, we can conclude that maximum contraction and maximum tissue thickness happen at (almost) the same time. A few outliers correspond to the ‘lowest collagen concentration’ simulations. Here, the difference in the tissue thickness between maximal contraction and maximal tissue thickness is 0.0143 cm, while, on average, the difference is 0.0008 cm.

The blue dots in Fig. 3 show the data points of the tissue thickness on day 365. The tissue thickness on day 365 varies between 0.164 and 0.198 cm, with an average deviation of 0.0037 cm. The wound boundary varies between 2.98 and 3.74 cm, with an average deviation of 0.076 cm.

The results change when we set the myofibroblast apoptosis to a significantly higher value of $\delta _m=0.06$/day. The corresponding values are shown in Table 3 in the Appendix 7. The collagen equilibrium again scores highest in sensitivity with a total score of 56. The following parameter is the signaling secretion rate ($k_c$), with a score of 28. So, there is a big jump in sensitivity, which is more significant than the jump for the low myofibroblast apoptosis simulations. These parameters score the highest in sensitivity for high and low myofibroblast apoptosis rates. Where the decay of signaling molecules plays an essential role in a low myofibroblast apoptosis rate, now fibroblast apoptosis ($\delta _n$) and collagen decay ($\delta _\rho$) do. Here, fibroblast apoptosis significantly affects the maximal tissue thickness, while collagen decay affects the tissue thickness after one year. When the myofibroblast apoptosis rate is high, the fibroblast distribution is of more importance. To effectively reduce TH$_{\max }$, we need to increase the (equilibrium) collagen concentration and the myofibroblast apoptosis rate and decrease the fibroblast apoptosis rate. Further, to effectively reduce TH$_{\text {end}}$, we need to increase the (equilibrium) collagen concentration and the signaling molecule and collagen decay rates and decrease the signaling secretion rate.

**Fig. 4:**
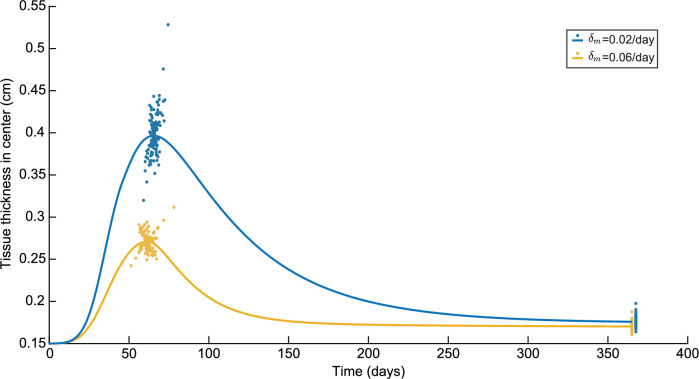
Variations in the tissue thickness in the center in a time of one year for both $\delta _m=0.02$/day and $\delta _m=0.06$/day. Here, the blue colors represent the results for $\delta _m=0.02$/day and the yellow colors represent the results to $\delta _m=0.06$/day

These new simulations also allow us to see to what extent the simulation results change the specific values of TH$_{\max }$ and TH$_{\text {end}}$, as seen in Fig. 4. In this figure, we slightly moved the results corresponding to $\delta _m=0.02$/day on day 365 such that the differences in variations are visible.

Here, the maximum tissue thickness is significantly lower for a high myofibroblast apoptosis rate with a difference of 0.13 cm in the control runs. The maximum tissue thickness for the low myofibroblast apoptosis is reached four days later. The difference of 0.0056 cm in tissue thickness on day 365 is less. Regarding the variations, for a high myofibroblast apoptosis rate, the variations on the maximum tissue thickness are $-$10.65% to +14.89%, and for a low apoptosis rate, $-$19.34% to +33.21%. On day 365, for a high myofibroblast apoptosis rate, the variations are $-$5.58% to +10.22%, and for a low apoptosis rate $-$6.77% to +12.40%. So, we see a greater spread of tissue thickness for the low apoptosis rate than for the high apoptosis rate, and the spread is more remarkable for the maximum tissue thickness than for the tissue thickness on day 365.

**Fig. 5:**
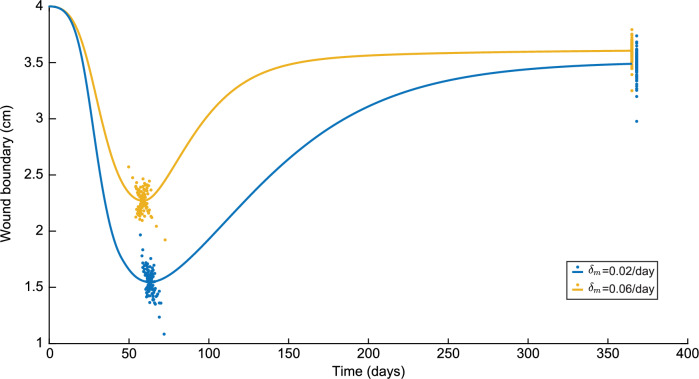
Variations in the wound boundary in a time of one year for both $\delta _m=0.02$/day and $\delta _m=0.06$/day. Here, the blue colors represent the results for $\delta _m=0.02$/day and the yellow colors represent the results to $\delta _m=0.06$/day

Next to the tissue thickness in the center, we can also see to what extent the simulation results change the specific values of the minimum boundary of the wound and the boundary of the wound on day 365, as seen in Figure 5. Again, we slightly moved the results corresponding to $\delta _m=0.02$/day on day 365 such that the differences in variations are visible.

Here, the minimum wound boundary is significantly lower for a low myofibroblast apoptosis rate with a difference of 0.72 cm in the control runs. As for the maximum tissue thickness, the minimum wound boundary for the low myofibroblast apoptosis is reached four days later. The difference of 0.1168 cm in the wound boundary on day 365 is less. Regarding the variations, for a high myofibroblast apoptosis rate, the variations on the minimum wound boundary are $-$15.35% to +13.25%, and for a low apoptosis rate, $-$29.97% to +27.08%. On day 365, for a high myofibroblast apoptosis rate, the variations are $-$9.88% to +5.20%, and for a low apoptosis rate $-$14.67% to +7.12%. So, similar to the tissue thickness, we see a greater spread of the wound boundary for the low apoptosis rate than for the high apoptosis rate, and the spread is more remarkable for the minimum wound boundary than for the wound boundary on day 365.

## Discussion

The model also shows a connection between contraction and hypertrophy. However, some patients only experience one of the two pathologies, such as hypertrophic scars without or with hardly any contraction and non-hypertrophic contractures. To this end, we might consider that portions of myofibroblasts either proliferate, produce collagen, or pull on the tissue, assuming a myofibroblast does not perform these processes simultaneously. The current model does not yet predict exclusively either of the two pathologies.

**Fig. 6:**
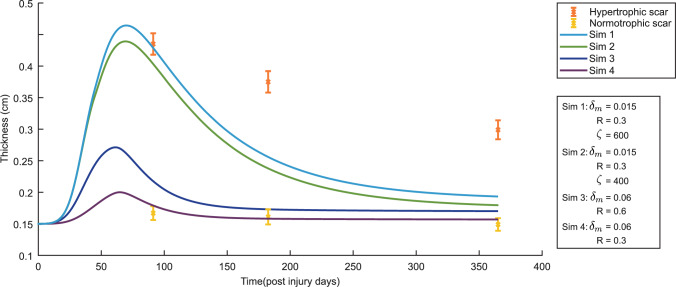
The evolution over time of the thickness of the dermal layer for different values for the apoptosis rate of myofibroblasts ($\delta _m$), the body force-inhibiting constant (*R*) and the rate of morphoelastic change ($\zeta$). The yellow error bars and the orange error bars represent clinical measurements of the thickness of normal scars and hypertrophic scars in human subjects at different time points after injury (Nedelec et al. 2014). Displayed are the means (with a cross sign) plus / minus one standard deviation

To assess the model’s ability to capture realistic tissue behavior, we compared the model’s predictions to clinical measurements of normal and hypertrophic scars (Nedelec et al. 2014), see Figure 6.

The model effectively captures the initial rapid increase in dermal thickness observed in the clinical data. However, the model predicts a subsequent decrease in thickness that is more rapid than the experimental measurements. The model also overestimates the peak thickness achieved, although the results are still within the error bars. In the case of a hypertrophic scar, this discrepancy suggests that the model’s current form may not fully capture the long-term processes that regulate dermal thickness during scar maturation. Potential factors contributing to this difference include the model’s simplified representation of tissue mechanics, the assumption of local equilibrium for MMP activity, and the exclusion of other cell types that play crucial roles in wound healing.

While the model exhibits limitations, such as its inability to fully capture persistent hypertrophy and the occasional decoupling of hypertrophy and contraction, these limitations are valuable insights gained through a detailed study of the model. A thorough analysis, including the sensitivity analysis presented here, is crucial for identifying these limitations, understanding the model’s capabilities, and guiding future model development. The acquired insights can and will be used to adjust the model where necessary so that the quality of the simulations will be improved.

This study, including the detailed sensitivity analysis, represents a crucial stepping stone toward more realistic and comprehensive models. The infinitesimal strain model is not sufficient to describe the slow decrease in skin thickness. With finite strain, we can better predict this gradual decrease. We need to do more simulations to quantify this in our future research.

In addition, hypertrophy depends highly on angiogenesis; therefore, we recommend including angiogenic factors in a future model. Including capillary cells, oxygen molecules, and macrophage cells is a good start.

The current model simplifies the mechanical properties of tissue by assuming isotropy, meaning they are uniform in all directions. While this allowed for manageable complexity in this initial study, it is important to acknowledge its limitations. Skin and scar tissue exhibit anisotropic behavior due to collagen fiber alignment, leading to direction-dependent mechanical responses, such as increased stiffness in the direction of fiber alignment. We chose this isotropic approach as a first step in developing a comprehensive framework for post-burn contraction and hypertrophy, where we focus on the interplay between biochemical signaling and mechanical forces. Furthermore, using an isotropic model provided a valuable baseline for comparison with future anisotropic models. Future research should prioritize incorporating anisotropy to enhance the model’s accuracy. This could be achieved by employing anisotropic constitutive relations, such as hyperelastic strain energy functions that account for collagen fiber orientation. Future models could also incorporate a variable that represents collagen fiber orientation. Addressing anisotropy will require careful consideration of parameter estimation and may necessitate experimental data on the skin’s directional mechanical properties.

During remeshing, we have to interpolate the data on the newly created nodes, for which interpolation errors are made. The interpolation errors will be dampened when the model satisfies the stability condition. However, remeshing is often needed for hypertrophy simulations compared to our previous simulations, where we previously only simulated contraction. For hypertrophy, the geometry of the computational domain is stretched: in the x-direction 10 cm and the y-direction 0.15 cm. When hypertrophy occurs, the corners of the triangles become sharp at a rapid pace. Isogeometric Analysis (IGA) can provide a solution since IGA is flexible in representing geometries and can approximate geometrically complex boundaries. In order to compare simulation results, the finite element code should then be converted to bilinear quadrilateral finite elements.

A complete wound model simulates in multiple dimensions. However, the computational load increases with dimension. Therefore, it would be necessary to apply intelligent techniques, such as programming in higher-level languages, such as C++, applying IGA, and reducing the computational domain. The goal is to eventually perform patient-specific simulations with associated probability distributions to provide treatment advice. To this end, it is essential to consider differences between patients and wounds and perform Monte Carlo simulations. Monte Carlo simulations in 3D will take a lot of time, and numerical simulations will not be lucrative. To this end, we also investigate the application of neural networks and recommend clever Monte Carlo techniques.

## Conclusion

One consequence of burns is the development of a hypertrophic scar that is thick, stiff, and red and can cause pain and itching in patients. Our ultimate objective is to optimize treatments that aim to prevent or at least minimize contracture and hypertrophy in severe burns.

To develop the mathematical model further and achieve this goal, we want to grasp its behavior and predictability strength. By varying input parameters for both high and low myofibroblast apoptosis, we show the relationship between the sensitivity of parameters for these two cases. We also present a possible spread in the input parameters so that the model gives realistic results.

While our biomorphoelastic model provides valuable insights into the early stages of post-burn contraction and hypertrophy, it relies on the assumption of infinitesimal strains. As suggested by the discrepancy between our model’s prediction of a rapid decrease in dermal thickness and the slower decrease observed in clinical data (as discussed earlier in the paper), this simplification limits its ability to capture the long-term tissue remodeling processes during scar maturation fully. The slow, gradual decrease in skin thickness observed clinically is likely better described by a finite strain formulation, which can account for more significant deformations and the nonlinear mechanical behavior of scar tissue over time. Therefore, future work will extend our model to incorporate finite strain theory to enhance its accuracy in predicting the later stages of scar development and maturation.

The (equilibrium) collagen concentration and the signaling molecule secretion rate are the most sensitive parameters for high and low myofibroblast apoptosis. We link their sensitivities to the stability of the model. When the collagen concentration is low, and the secretion rate of signaling molecules is high, the parameter values are closer to the (linear) stability limit. In such cases, the model tends to give oscillatory results and has difficulty accounting for these oscillations, resulting in frequent remeshing operations and high sensitivity for these parameters. These two parameters account for 28.8% and 30.11% sensitivity for low and high myofibroblast apoptosis, respectively. However, in the first 58% of the sensitivity (from high to low) for low apoptosis, we only see parameters belonging to collagen and signaling molecules.

In contrast, these parameters appear earlier in the sensitivity for high apoptosis. In other words, the role of fibroblasts is less significant than the role of signaling molecules and collagen when myofibroblasts survive. In contrast, fibroblast apoptosis plays a relatively significant role when myofibroblast apoptosis is high.

Our findings suggest increasing the collagen concentration and stimulating myofibroblast apoptosis to counteract hypertrophy effectively. Less effective, but still recommended, strategies include stimulating the survival of fibroblasts and limiting possible stress exerted on them. The collagen concentration could be increased using a skin graft or collagen-rich skin substitutes, and the stress could be reduced by limiting its influencing factors or splinting. In the latter case, we consider limiting environmental mechanical forces. Stimulating the survival of fibroblasts should be further investigated to prevent the method from allowing myofibroblasts to survive.

## Data Availability

No datasets were generated or analyzed during the current study.

## Appendix

Table 1 shows the average parameter values used in the sensitivity analysis.

**Table 1:** Average parameter values used in the sensitivity analysis

Parameter	Value	Dimension
$\overline{N}$	$10^4$	cells/cm$^3$
$\overline{\rho }$	$1.125\times 10^{-1}$	g/cm$^3$
$D_n$	$10^{-7}$	cm$^5$/(cells day)
$D_c$	$2.9\times 10^{-3}$	cm$^2$/day
$\chi$	$2\times 10^{-3}$	cm$^5$/(g day)
$r_n$	$9.24\times 10^{-1}$	cm$^{3q}$/(cells$^q$ day)
$r_m$	$9.24\times 10^{-1}$	cm$^{3q}$/(cells$^q$ day)
$r^{\max }$	2	–
$k^{\max }$	10	–
$a_c^I$	$10^{-8}$	g/cm$^3$
$a_c^{II}$	$10^{-8}$	g/cm$^3$
$a_c^{III}$	$2\times 10^8$	cm$^3$/g
$a_c^{IV}$	$10^{-9}$	g/cm$^3$
$\kappa$	$10^{-6}$	cm$^3$/cells
$\delta _n$	$2\times 10^{-2}$	/day
$\delta _c$	$5\times 10^{-4}$	g/cm$^3$
$\delta _\rho$	$6\times 10^{-6}$	cm$^6$/(cells g day)
$k_1$	$5.4\times 10^6$	cm$^3$/(g day)
$k_c$	$4\times 10^{-13}$	g/(cells day)
$\eta ^I$	2	–
$\eta ^{II}$	1	–
$\rho _t$	1.09	g/cm$^3$
$\mu _1$	$2\times 10^2$	(N day)/cm$^2$
$\mu _2$	$2\times 10^2$	(N day)/cm$^2$
*E*	$3.2\times 10$	N/((g cm)$^{1/2}$)
$\zeta$	$4\times 10^2$	cm$^6$/(cells g day)
$\xi$	$2\times 10^{-3}$	(N g)/(cells cm$^2$)
*R*	$3\times 10^{-1}$	g/cm$^3$
$\tilde{c}$	$10^{-8}$	g/cm$^3$

Some values depend on other values or are fixed. These are $\nu =4.9\times 10^{-1}$, $q=(\log (\delta _n)-\log (r_n\cdot (1-\kappa \cdot \overline{N})))/\log (\overline{N})$, and $k_\rho =\delta _\rho \cdot \overline{\rho }^2$. The myofibroblast apoptosis rate is either $\delta _m=0.06$/day or $\delta _m=0.02$/day. For an extensive overview of the choice of parameter values, we refer to Egberts et al. (July 2021)

Table 2 shows the sensitivity values in terms of the *z*-scores for all varied parameter values given a low myofibroblast apoptosis value. In the last column, we rounded the sum of the values to the nearest integer.

**Table 2:** Sensitivity of the varied parameter values in terms of *z*-scores for a low myofibroblast apoptosis value

Param	Dimension	$\mathcal {S}^{\text {TH}_\text {max}}$	$\mathcal {S}^{\text {TH}_\text {day}}$	$\mathcal {S}^{\text {TH}_\text {end}}$	$\mathcal {S}^\text {total}$
$\overline{\rho }$	g/cm$^3$	19.437	14.657	19.62	54
$k_c$	g/(cells day)	9.484	14.315	13.63	37
$\delta _c$	g/cm$^3$	6.268	11.571	10.07	28
$a_c^{III}$	cm$^3$/g	6.078	12.546	7.191	26
$k_{\rho }^{max}$	–	7.27	5.302	7.759	20
$\delta _\rho$	cm$^6$/(cells g day)	6.517	3.954	8.75	19
$\xi$	(N g)/(cells cm$^2$)	10.426	1.335	6.754	18
$\eta ^{II}$	–	2.655	9.34	5.092	17
$\delta _n$	/day	8.4	3.932	2.625	15
*R*	g/cm$^3$	6.492	2.686	5.263	14
$r_m$	cm$^{3q}$/(cells$^q$ day)	7.263	2.484	2.918	13
$\eta ^I$	–	0.265	7.532	3.661	11
$r^{max}$	–	6.211	3.19	1.512	11
$\overline{N}$	cells/cm$^3$	4.954	3.957	1.885	11
$\zeta$	cm$^6$/(cells g day)	2.457	2.088	6.031	11
$r_n$	cm$^{3q}$/(cells$^q$ day)	4.657	5.013	0.507	10
$\mu _1$	(N day)/cm$^2$	1.385	5.82	1.954	9
*E*	N/((g cm)$^{1/2}$)	5.937	1.799	0.682	8
$\delta _m$	/day	3.402	2.523	0.464	6
$\tilde{c}$	g/cm$^3$	1.958	0.847	2.809	6
$a_c^I$	g/cm$^3$	0.72	3.502	0.861	5
$\chi$	cm$^5$/(g day)	1.051	1.616	2.164	5
$a_c^{IV}$	g/cm$^3$	1.409	1.807	1.338	5
$k_1$	cm$^3$/(g day)	0.547	2.088	1.795	4
$D_c$	cm$^2$/day	1.107	1.616	1.341	4
$\kappa$	cm$^3$/cells	1.024	1.616	1.283	4
$\mu _2$	(N day)/cm$^2$	1.091	1.616	1.125	4
$\rho _t$	g/cm$^3$	1.034	1.616	1.161	4
$D_n$	cm$^5$/(cells day)	0.879	1.616	1.173	4
$a_c^{II}$	g/cm$^3$	0.783	1.059	1.258	3

The third, fourth and fifth columns show the *z*-scores on the maximum of the tissue thickness in the center of the wound $\mathcal {S}^{\text {TH}_\text {max}}$, the day on which the maximum tissue thickness is reached $\mathcal {S}^{\text {TH}_\text {day}}$ and the maximum of the tissue thickness in the center of the wound on day 365 $\mathcal {S}^{\text {TH}_\text {end}}$. The last column shows the total of the *z*-scores $\mathcal {S}^\text {total}$

Table 3 shows the sensitivity values in terms of the *z*-scores for all varied parameter values for a high myofibroblasts apoptosis value. In the last column, we rounded the sum of the values to the nearest integer.

**Table 3:** Sensitivity of the varied parameter values in terms of *z*-scores for a high myofibroblast apoptosis value

Param	Dimension	$\mathcal {S}^{\text {TH}_\text {max}}$	$\mathcal {S}^{\text {TH}_\text {day}}$	$\mathcal {S}^{\text {TH}_\text {end}}$	$\mathcal {S}^\text {total}$
$\overline{\rho }$	g/cm$^3$	15.8	19.334	20.389	56
$k_c$	g/(cells day)	4.963	10.258	12.909	28
$\delta _n$	/day	11.107	7.497	4.511	23
$\delta _\rho$	cm$^6$/(cells g day)	6.423	7.917	8.731	23
$\xi$	(N g)/(cells cm$^2$)	12.108	1.928	6.984	21
$k_{\rho }^{max}$	–	5.92	7.18	7.886	21
$r^{max}$	–	8.776	5.854	3.697	18
$\delta _c$	g/cm$^3$	2.009	6.672	9.331	18
*R*	g/cm$^3$	8.102	4.032	5.251	17
$r_m$	cm$^{3q}$/(cells$^q$ day)	9.345	3.75	4.052	17
$a_c^{III}$	cm$^3$/g	2.166	8.559	5.934	17
$\overline{N}$	cells/cm$^3$	6.667	5.972	2.981	16
$\delta _m$	/day	7.28	6.797	1.416	15
$r_n$	cm$^{3q}$/(cells$^q$ day)	6.114	5.931	1.36	13
$\zeta$	cm$^6$/(cells g day)	2.501	2.619	6.216	11
$\eta ^{II}$	–	0.845	5.044	3.742	10
*E*	N/((g cm)$^{1/2}$)	7.013	0.422	1.54	9
$\mu _1$	(N day)/cm$^2$	2.323	3.914	1.831	8
$\eta ^I$	–	2.619	1.237	2.959	7
$a_c^I$	g/cm$^3$	1.476	4.422	0.459	6
$k_1$	cm$^3$/(g day)	0.539	4.263	1.253	6
$\tilde{c}$	g/cm$^3$	1.442	1.928	2.483	6
$a_c^{IV}$	g/cm$^3$	1.117	1.928	1.485	5
$\mu _2$	(N day)/cm$^2$	0.941	1.928	1.251	4
$\chi$	cm$^5$/(g day)	0.938	1.928	1.249	4
$\kappa$	cm$^3$/cells	0.971	1.928	1.209	4
$\rho _t$	g/cm$^3$	0.906	1.928	1.196	4
$D_c$	cm$^2$/day	0.802	1.928	1.296	4
$D_n$	cm$^5$/(cells day)	0.878	1.928	1.2	4
$a_c^{II}$	g/cm$^3$	0.101	0.59	1.846	3

The third, fourth and fifth columns show the *z*-scores on the maximum of the tissue thickness in the center of the wound $\mathcal {S}^{\text {TH}_\text {max}}$, the day on which the maximum tissue thickness is reached $\mathcal {S}^{\text {TH}_\text {day}}$, and the maximum of the tissue thickness in the center of the wound on day 365 $\mathcal {S}^{\text {TH}_\text {end}}$. The last column shows the total of the *z*-scores $\mathcal {S}^\text {total}$

## References

[CR1] Barrientos S, Stojadinovic O, Golinko M, Brem H, Tomic-Canic M (2008) Perspective article: growth factors and cytokines in wound healing. Wound Repair Regeneration 16(5):585–601 19128254 10.1111/j.1524-475X.2008.00410.x

[CR2] Baum C, Arpey C (2006) Normal cutaneous wound healing: clinical correlation with cellular and molecular events. Dermatol Surg 31(6):674–686 10.1111/j.1524-4725.2005.31612 15996419

[CR3] Boon W, Koppenol D, Vermolen F (2016) A multi-agent cell-based model for wound contraction. J Biomech 49(8):1388–1401 26805459 10.1016/j.jbiomech.2015.11.058

[CR4] Dallon J, Sherrat J, Maini P (2001) Modeling the effects of transforming growth factor- on extracellular matrix alignment in dermal wound repair. Wound Repair Regeneration 9(4):278–286 11679136 10.1046/j.1524-475x.2001.00278.x

[CR5] Egberts G, Desmoulière A, Vermolen F, van Zuijlen P (2022) Sensitivity of a two-dimensional biomorphoelastic model for post-burn contraction. Biomech Model Mechanobiol 22(1):105–121 36229698 10.1007/s10237-022-01634-w PMC9957927

[CR6] Egberts G, Vermolen F, van Zuijlen P (2021) Sensitivity and feasibility of a one-dimensional morphoelastic model for post-burn contraction. Biomech Model Mechanobiol 20(6):2147–2167 34331622 10.1007/s10237-021-01499-5 PMC8595192

[CR7] Egberts G, Vermolen F, van Zuijlen P (2023) Stability of a two-dimensional biomorphoelastic model for post-burn contraction. J Math Biol 86(4):59 36964257 10.1007/s00285-023-01893-w PMC10038978

[CR8] El Hadidy M, Tesauro P, Cavallini M, Colonna M, Rizzo F, Signorini M (1994) Contraction and growth of deep burn wounds covered by non-meshed and meshed split thickness skin grafts in humans. Burns 20(3):226–228 8054134 10.1016/0305-4179(94)90187-2

[CR9] Hall C (2008) Modelling of some biological materials using continuum mechanics. PhD thesis, Queensland University of Technology

[CR10] Ivanoff J, Talme T, Sundqvist K (2005) The role of chemokines and extracellular matrix components in the migration of T lymphocytes into three-dimensional substrata. Immunology 114(1):53–62 15606795 10.1111/j.1365-2567.2004.02005.x PMC1782061

[CR11] Koppenol D (2017) Biomedical implications from mathematical models for the simulation of dermal wound healing. PhD thesis, Delft University of Technology

[CR12] Koppenol D, Vermolen F (2017) Biomedical implications from a morphoelastic continuum model for the simulation of contracture formation in skin grafts that cover excised burns. Biomech Model Mechanobiol 16(4):1187–1206 28181018 10.1007/s10237-017-0881-y PMC5511621

[CR13] Koppenol D, Vermolen F, Niessen F (2017) A mathematical model for the simulation of the formation and the subsequent regression of hypertrophic scar tissue after dermal wounding. Biomech Model Mechanobiol 16(1):15–32 27229739 10.1007/s10237-016-0799-9 PMC5285433

[CR14] Lindner D, Zietsch C, Becher P, Schulze K, Schultheiss H, Tschöpe C, Westermann D (2012) Differential expression of matrix metalloproteases in human fibroblasts with different origins. Biochem Res Int 2012:1–10 10.1155/2012/875742 PMC3303709 22500233

[CR15] Mast B, Schultz G (1996) Interactions of cytokines, growth factors, and proteases in acute and chronic wounds. Wound Repair Regeneration 4(4):411–420 17309691 10.1046/j.1524-475X.1996.40404.x

[CR16] Murray J (2011) Mathematical Biology II. Springer, New York

[CR17] Nedelec B, Correa JA, de Oliveira A, LaSalle L, Perrault I (2014) Longitudinal burn scar quantification. Burns 40(8):1504–1512 24703337 10.1016/j.burns.2014.03.002

[CR18] Olsen L, Sherratt JA, Maini PK (1995) A mechanochemical model for adult dermal wound contraction and the permanence of the contracted tissue displacement profile. J Theor Biol 177(2):113–128 8558902 10.1006/jtbi.1995.0230

[CR19] Overall C, Wrana J, Sodek J (1991) Transcriptional and post-transcriptional regulation of 72-kda gelatinase/ type IV collagenase by transforming growth factor-beta in human fibroblasts. J Biol Chem 266(21):14061–14071 1649834

[CR20] Pierce GF (2001) Inflammation in nonhealing diabetic wounds. Am J Pathol 159(2):399–403 11485896 10.1016/S0002-9440(10)61709-9 PMC1850546

[CR21] Postlethwaite A, Keski-Oja J, Moses H, Kang A (1987) Stimulation of the chemotactic migration of human fibroblasts by transforming growth factor beta. J Exp Med 165(1):251–256 3491869 10.1084/jem.165.1.251 PMC2188256

[CR22] Ramtani S (2004) Mechanical modelling of cell/ECM and cell/cell interactions during the contraction of a fibroblast-populated collagen microsphere: theory and model simulation. J Biomech 37(11):1709–1718 15388313 10.1016/j.jbiomech.2004.01.028

[CR23] Ramtani S, Fernandes-Morin E, Geiger D (2002) Remodeled-matrix contraction by fibroblasts: numerical investigations. Comput Biol Med 32(4):283–296 11931865 10.1016/s0010-4825(02)00018-5

[CR24] Sternlicht M, Werb Z (2001) How matrix metalloproteinases regulate cell behavior. Annu Rev Cell Dev Biol 17(1):463–516 11687497 10.1146/annurev.cellbio.17.1.463 PMC2792593

[CR25] Tomasek J, Gabbiani G, Hinz B, Chaponnier C, Brown R (2002) Myofibroblasts and mechano-regulation of connective tissue remodelling. Nat Rev Mol Cell Biol 3(5):349–363 11988769 10.1038/nrm809

[CR26] Vande Berg J, Rudolph R, Poolman W, Disharoon D (1989) Comparative growth dynamics and actin concentration between cultured human myofibroblasts from granulating wounds and dermal fibroblasts from normal skin. Lab Invest 61(5):532–538 2811301

[CR27] Vaughan M, Odejimi T, Morris T, Sawalha D, Spencer C (2014) A new bioassay identifies proliferation ratios of fibroblasts and myofibroblasts. Cell Biol Int 38(8):981–986 24764319 10.1002/cbin.10289

